# Alternative exon skipping biases substrate preference of the deubiquitylase USP15 for mysterin/RNF213, the moyamoya disease susceptibility factor

**DOI:** 10.1038/srep44293

**Published:** 2017-03-09

**Authors:** Yuri Kotani, Daisuke Morito, Kenshiro Sakata, Shiori Ainuki, Munechika Sugihara, Tomohisa Hatta, Shun-ichiro Iemura, Seiji Takashima, Tohru Natsume, Kazuhiro Nagata

**Affiliations:** 1 Faculty of Life Sciences, Kyoto Sangyo University, 603-8555, Japan; 2 Institute for Protein Dynamics, Kyoto Sangyo University, 603-8555, Japan; 3 CREST, Japan Science and Technology Agency, 332-0012, Japan; 4 Biomedicinal Information Research Center, National Institute of Advanced Industrial Science and Technology, 135-0064, Japan; 5 Graduate School of Medicine, Osaka University, 565-0871, Japan

## Abstract

The deubiquitylating enzyme USP15 plays significant roles in multiple cellular pathways including TGF-β signaling, RNA splicing, and innate immunity. Evolutionarily conserved skipping of exon 7 occurs during transcription of the mRNAs encoding USP15 and its paralogue USP4, yielding two major isoforms for each gene. Exon 7 of USP15 encodes a serine-rich stretch of 29 amino acid residues located in the inter-region linker that connects the N-terminal putative regulatory region and the C-terminal enzymatic region. Previous findings suggested that the variation in the linker region leads to functional differences between the isoforms of the two deubiquitylating enzymes, but to date no direct evidence regarding such functional divergence has been published. We found that the long isoform of USP15 predominantly recognizes and deubiquitylates mysterin, a large ubiquitin ligase associated with the onset of moyamoya disease. This observation represents the first experimental evidence that the conserved exon skipping alters the substrate specificity of this class of deubiquitylating enzymes. In addition, we found that the interactomes of the short and long isoforms of USP15 only partially overlapped. Thus, USP15, a key gene in multiple cellular processes, generates two functionally different isoforms via evolutionarily conserved exon skipping.

Protein mono- and poly-ubiquitylation regulates multiple cellular phenomena, including selective protein degradation in the cytosol^1^. The reverse of these reactions, deubiquitylation, involves cleavage of ubiquitin–substrate and ubiquitin–ubiquitin bonds. The enzymes that catalyze this process and balance the ubiquitin signaling *in vivo* are collectively termed deubiquitylating enzymes (DUBs)^2^,^3^. All known DUBs are either cysteine proteases or metalloproteases, and are currently classified into five subgroups based on the structures of their catalytic centers. Ubiquitin-specific proteases (USPs), which constitute the largest subgroup of known DUBs, are papain-like cysteine proteases and are characterized by a binary catalytic center consisting of two consensus elements called the His and Cys boxes^2^,^3^.

Three of the more than 80 known human USPs, USP4, 11, and 15, constitute a more closely related subgroup^4^. These USPs have independent but partly overlapping substrate specificities related to multiple cellular phenomena including TGFβ signaling, RNA splicing, and innate immunity^5^,^6^,^7^,^8^,^9^,^10^,^11^. In mice, single knockout of either USP4 or USP15 does not interfere with viability and normal development, whereas the double knockout results in embryonic lethality, suggesting that these proteins have redundant functions^4^. Structurally, all three of these USPs share a characteristic domain organization consisting of a domain present in USPs (DUSP), two ubiquitin-like (UBL) motifs, and a zinc finger domain, in addition to the binary catalytic center (Fig. 1). The C-terminal region of these three USPs includes a zinc finger domain, one of the UBL motifs, and the binary core that directly catalyzes the deubiquitylation reaction^12^,^13^, whereas the N-terminal region includes a DUSP and the other UBL motif, which is also indispensable for deubiquitylation^8^,^9^,^14^. The N-terminal region presumably contributes to regulation of deubiquitylation and/or substrate recognition. In addition, the linker region connecting the N- and the C-terminal regions plays a role in deubiquitylation^14^, but the precise molecular roles of the N-terminal and linker regions remain elusive.

USP4 and 15 each have two evolutionarily conserved alternate isoforms^4^,^15^,^16^, of which biological functions have been assumed to be different. Alternative skipping of exon 7 results in expression of the short isoforms of USP4 and 15^17^, which have shorter linker regions between the N- and C-regions but retain deubiquitylation activities comparable to those of the long isoforms^5^,^14^. The two isoforms of USP4 have clearly different subcellular distributions (long: predominantly in the cytosol; short: throughout the nucleus and cytosol) under identical experimental conditions, although the mechanisms underlying this difference remain unknown^17^. A previous study focusing on the mechanism of the exon skipping identified a specific element that causes the two alternative isoforms to be generated during the maturation process of USP4 mRNA^17^. However, this element is not conserved in USP15, even though the USP15 mRNA undergoes similar alternative skipping of exon 7, suggesting that over the course of evolution USP15 acquired an independent mechanism for skipping of exon 7. Both isoforms of USP4 and 15 are expressed at similar levels in mammals^16^,^17^, suggesting moderate rates of exon skipping, while the ratio of the two isoforms of USP4 is specifically altered in Paget’s disease of bone^18^, suggesting that the ratio is of pathological relevance. Together, these findings suggest that the isoforms of this class of USPs have physiologically distinct functions; to date, however, the nature of any such functional difference remains unknown.

We discovered functional bias in the isoforms of USP15 during an exploration of the biological relevance of a novel protein. In previous work, we isolated a key gene involved in the cryptogenic cerebrovascular disorder moyamoya disease^19^, in which progressive stenosis and occlusion in a limited region of cerebral arteries ultimately cause ischemic stroke, cerebral infarction, and development of fragile collateral capillary vessels^20^. Although the pathogenesis of moyamoya disease remains largely unknown, the fact that some patients have a family history suggested that genetic factor(s) play an important role in pathogenesis^21^. We found that a rare missense single-nucleotide polymorphism (SNP) in a novel gene with unknown function increased the risk of moyamoya disease by over 100-fold^19^. We cloned this gene and named it mysterin (moyamoya steno-occlusive disease-associated AAA+ and RING finger protein; also known as RNF213)^19^,^22^. The mysterin gene encodes a large (591 kDa) intracellular protein with a unique domain organization consisting of two active AAA+ ATPase modules and a RING finger ubiquitin ligase domain. Suppression of mysterin expression results in abnormal angiogenesis and myogenesis in zebrafish, suggesting that the disease-associated SNP interferes with the physiological role of mysterin in angiogenesis, resulting in moyamoya disease^19^. To further elucidate the cellular function of mysterin, we searched for substrates and/or cofactors using liquid chromatography–tandem mass spectrometry (LC-MS/MS), ultimately identifying USP15.

Here, we report the first example of isoform-specific bias in substrate preference of USP15. Biochemical evaluation of the association between USP15 and mysterin revealed that mysterin is predominantly recognized by the long isoform of USP15. Moreover, the USP15 long isoform potently deubiquitylated mysterin and maintained its basal expression level, indicating that alternative exon skipping biases the deubiquitylation substrate preference of USP15. In addition, we performed an extensive LC-MS/MS analysis of USP15-binding proteins and identified multiple isoform-specific binding proteins, suggesting that the two isoforms of USP15 play roles in distinct proteome networks and have overlapping but independent cellular activities *in vivo*.

## Results

### The long isoform of USP15 predominantly recognizes mysterin

To elucidate the cellular function of the moyamoya disease-associated protein mysterin, we sought to identify mysterin-binding proteins, reasoning that such proteins would include cofactors and/or substrates of mysterin. To this end, we transiently overexpressed mysterin harboring a FLAG tag at its C-terminus (mysterin-FLAG) in HEK293T human embryonic kidney cells. We precipitated mysterin-FLAG from cell lysates using anti-FLAG antibody and performed LC-MS/MS analysis on total co-precipitates without an isolation process such as SDS-PAGE. Four positive hits, including USP15, LYAR, hnRNPK, and IP3 receptor isoform 3 (IP3R3) in addition to mysterin itself, were reproducibly identified in four trials. In a replication study, we transiently expressed each candidate mysterin-binding protein in HEK293T cells, immunoprecipitated using specific antibodies, and then immunoblotted for mysterin. In this analysis, USP15 and LYAR were detectably physically associated with mysterin, whereas the other two proteins were not (Fig. 2A and B). However, LYAR is a factor that functions in the nucleolus^23^,^24^, whereas mysterin is predominantly distributed in the cytosol. Accordingly, although we do not exclude the possibility that LYAR or the other two proteins are functionally associated with mysterin despite their weaker binding and/or inconsistent subcellular distribution under normal condition, we focused on USP15 for the remainder of this study. It is worth noting that mysterin is often detected as multiple bands in immunoblot analysis (see difference in Fig. 2A and B). Independent immunoblot trials even with an identical mysterin protein sample sometimes result in different appearances like those seen in Fig. 2A and B. Thus, we think that it is not caused by any difference in biological conditions but possibly caused by exceptionally large molecular size of mysterin and minor procedural effect in the protein electrophoresis.

We obtained cDNAs encoding both isoforms of USP15 from HEK293 cells by reverse transcription–polymerase chain reaction (RT-PCR). To our surprise, the isoform of USP15 with longer linker region (hereafter USP15L) had significantly stronger affinity for mysterin than the other isoform with the shorter linker (hereafter USP15S; Fig. 2C). Similar alternative skipping in the linker region is observed in USP4, a paralogue of USP15, and the two isoforms of USP4 have different subcellular distributions (see Introduction for details). When we examined the subcellular distributions of the isoforms of USP15, we found that both isoforms were typically localized in the cytosol, along with mysterin, while a minority of cells showed faint signal of USP15S also in the nucleus (Fig. 2D,E, and Supplementary Fig. S1). Thus, USP15L binds more strongly to mysterin than USP15S, and this effect is primarily not a result of isoform-specific differences in subcellular distribution.

### The long isoform of USP15 deubiquitylates mysterin

We hypothesized that the alternative exon skipping of USP15 biases its substrate preference. To test this idea, we investigated whether USP15 has an isoform-specific preference for mysterin as a deubiquitylation substrate. For this purpose, the long and short isoforms of USP15 were individually co-expressed in HEK293T cells along with mysterin harboring three tandem FLAG tags at its C-terminus (mysterin-3FLAG). Mysterin-3FLAG was precipitated using FLAG affinity beads, and its ubiquitylation state was examined by immunoblotting using anti-ubiquitin antibody. Overexpression of USP15L significantly decreased the polyubiquitin chain signal associated with mysterin-3FLAG, whereas overexpression of USP15S did not, suggesting that USP15L has the stronger deubiquitylation activity against mysterin (Fig. 3A). The polyubiquitin signal detected here is due to ubiquitylated mysterin protein *per se* but not to potentially coimmunoprecipitated proteins, since similar polyubiquitylation is detected even after dissociation of potential non-covalent protein interaction by incubation with 0.5% SDS (Supplementary Fig. 2). Mutations in the enzymatically essential Cys box and zinc finger domain of USP15L abrogated its deubiquitylation activity against mysterin (Fig. 3B), but did not affect the association between USP15L and mysterin (Fig. 3C), supporting the idea that USP15L deubiquitylates its substrate mysterin via its intrinsic enzymatic activity. Moreover, the USP15L mutant increased the level of mysterin-associated polyubiquitin, indicating that, at least in HEK293T cells, this mutant has a dominant-negative effect on the endogenous deubiquitylation activities against mysterin. Together, our data demonstrate that mysterin is a substrate of USP15, and that the conserved skipping of exon 7 significantly decreases its specific affinity for mysterin.

### The long isoform of USP15 contributes to stable expression of mysterin

Previous studies demonstrated that USP15 removes K48-linked polyubiquitin chains and thereby protects its substrates against proteasomal protein degradation triggered by K48-linked polyubiquitylation^5^,^25^. We investigated whether this would also be the case for deubiquitylation of mysterin by USP15L. Immunoprecipitation of mysterin-3FLAG followed by immunoblotting of K48-linked polyubiquitin revealed that mysterin was indeed modified with K48-linked polyubiquitin, which was removed by wild-type USP15L but accumulated in cells expressing the enzymatic mutant (Fig. 4A). Two further observations supported our prediction that the deubiquitylation of mysterin is inversely correlated with polyubiquitylation-induced proteasomal degradation. First, proteasome inhibitor treatment resulted in accumulation of the polyubiquitylated species of mysterin, whereas this species disappeared upon overexpression of USP15L (Fig. 4B). Second, the enzymatic mutant of USP15L destabilized mysterin protein in HEK293T cells, presumably by increasing the polyubiquitylation of mysterin through the mutant’s dominant-negative effect on endogenous mysterin-deubiquitylation activity in HEK293T cells (Fig. 4C and D), while the mutant of USP15S did not affect the stability of mysterin (Supplementary Fig. S3A and B). Thus, USP15L preferentially removes K48-linked polyubiquitin from mysterin and maintains its basal expression level in the cell.

### The linker region is necessary but not sufficient for substrate recognition

How does the alternative variation in the linker region bias the affinity of USP15 for mysterin? Although the substrate-binding domain of USP15 has not been definitely identified, one simple model is that the linker region directly mediates mysterin binding. We evaluated the roles of structural regionss and domains in USP15, including the long linker region, in the mysterin-binding process using five truncated mutants of USP15, schematically represented in Fig. 5A. Each variant, harboring three tandem FLAG tags at its C-terminal end, was transiently overexpressed in HEK293T cells. Each USP15 variant was precipitated using FLAG affinity beads, and co-precipitated endogenous mysterin was detected by immunoblotting with anti-mysterin antibody. The results revealed that both the N- and C-terminal regions are essential for mysterin binding (Fig. 5B). However, truncated mutants harboring the long linker region, such as ΔC1, ΔC2, ΔN2, and ΔN3 (see Fig. 5A), failed to bind to mysterin, indicating that the long linker is essential but not sufficient for the physical association with mysterin. The DUSP domain and C-region were also necessary but not sufficient for binding (Fig. 5B, lanes 3 and 8). These results are consistent with a model in which the DUSP domain and C-terminal region cooperatively clasp mysterin, and this cooperativity is coordinated by the linker region in an isoform-specific manner (see Discussion).

### The two USP15 isoforms function in different protein networks

To further characterize the functional divergence of the two major isoforms of USP15, we performed an interactome analysis on each isoform, based on the expectation that each interactome will include substrates and/or cofactors specific for each isoform. We transiently overexpressed the long and short isoforms of USP15, C-terminally tagged with three tandem FLAG epitopes, in HEK293T cells; precipitated them from cell lysate using anti-FLAG antibody; and performed LC-MS/MS analysis on total co-precipitates without isolation. Positive hits reproducibly obtained in all four trials are shown in Fig. 6 in Venn diagram format. Proteins shown in blue and red are substrates and/or cofactors previously identified by interactome analysis^26^ or experimentally evaluated in previous studies^9^,^27^,^28^, respectively. Although faint physical interaction of mysterin with the short isoform of USP15 was detected in immunoblot analysis (Fig. 2C), the LC-MS/MS analysis identified mysterin only in the long-interactome (Fig. 6), which is possibly due a difference between detection limits or sensitivities of the two experimental procedures. We do not distinguish between substrates and cofactors in these interactomes. However, the partial (but not complete) overlap between the interactome patterns suggests that the functional diversity between the two evolutionarily conserved isoforms of USP15 is not limited to deubiquitylation of mysterin.

## Discussion

**Figure 1:**
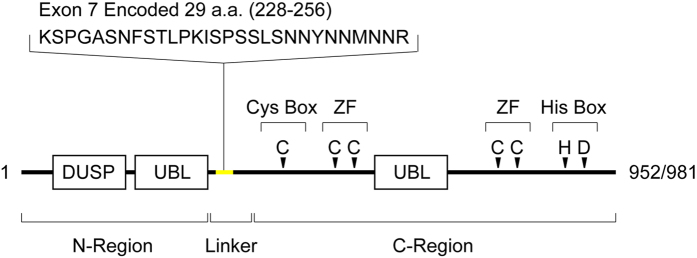
Primary structure of USP15. The long and short isoforms of USP15 consist of 952 and 981 amino acid residues, respectively. The difference between them, the 29-amino acid element encoded by exon 7, is in the inter-region linker between the N- and C-terminal domain clusters. The N-region contains a domain present in USP (DUSP) and a ubiquitin-like motif (UBL), whereas the C-region contains another UBL motif, the enzymatically essential Cys and His boxes, and the enzymatically essential zinc finger (ZF) domain. Four essential ligand cysteines constituting a single ZF are separated by the second UBL domain in the primary structure. The organization of these motifs is conserved among USP4, USP15, and USP11.

Alternative skipping of exon 7 in USP15 resulted in deletion of a short element consisting of 29 amino acids in the inter-region linker of USP15 (Fig. 1), which significantly decreased the affinity and deubiquitylation activity of USP15 toward mysterin. Previous studies demonstrated that even the short isoforms of USP15 and its paralogue USP4 retain deubiquitylation activities, indicating that the exon skipping does not abrogate the enzymatic activities of these USPs^5^,^14^. Instead, it is more likely that the exon skipping modulates the substrate specificity of this class of USPs. Our LC-MS/MS analysis revealed that the interactome patterns of the two isoforms of USP15 overlapped partially, but not completely, i.e., some potential substrates are recognized preferably by the short or long isoform of USP15.

**Figure 2:**
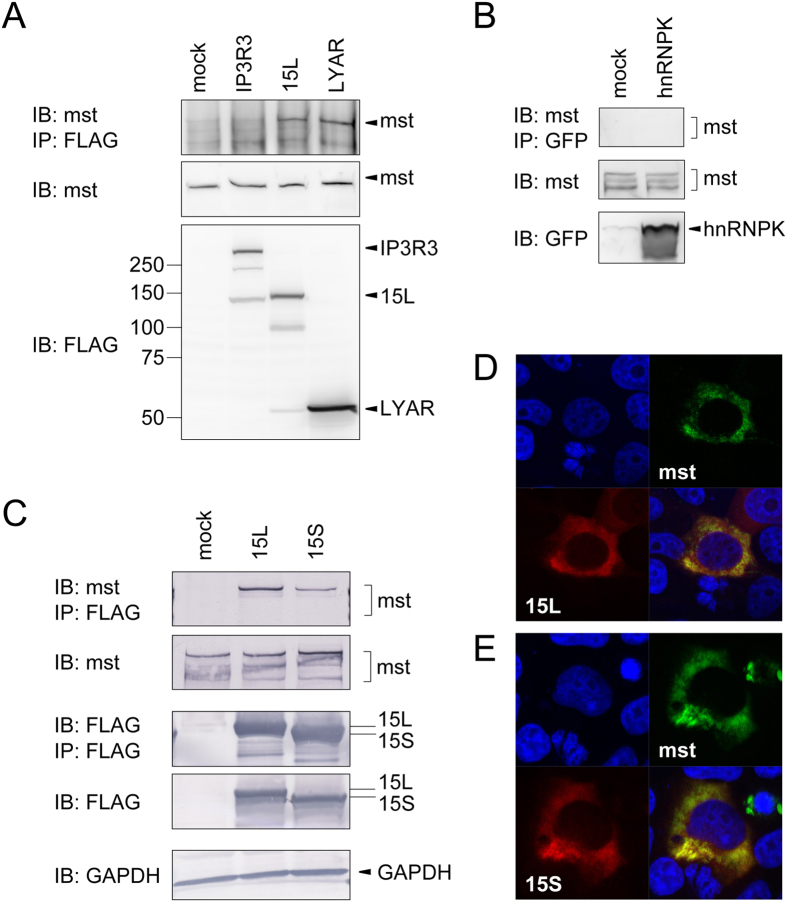
Long isoform of USP15 predominantly binds to mysterin. (**A**) IP3R3, USP15 (long isoform, 15 L), and LYAR harboring a FLAG epitope tag at their C termini were expressed in HEK293T cells and precipitated using anti-FLAG affinity beads. Endogenous mysterin (591 kDa) co-precipitated with the bait protein was detected with anti-mysterin antibody in the high-molecular weight region of the SDS polyacrylamide gel (top panel). Basal expression levels of mysterin and bait proteins in the cell were detected in the lower panels. Standard molecular sizes are shown on the left side; the same marker was used for all subsequent experiments. Full-length blots are presented in Supplementary Fig. 4A. (**B**) hnRNPK harboring an EGFP tag at its N terminus was expressed and immunoprecipitated under the same conditions, but no co-immunoprecipitated mysterin was detected. Full-length blots are presented in Supplementary Fig. 4B. (**C**) Long and short (15S) isoforms of USP15 harboring a FLAG epitope tag at their C termini (USP15L-FLAG and USP15S-FLAG) were transiently expressed and precipitated. Endogenous mysterin preferentially co-precipitated with the long isoform. Full-length blots are presented in Supplementary Fig. 4C. (**D**,**E**) Long and short isoforms of USP15 harboring Myc epitope tag at their C-terminus (USP15L-Myc and USP15S-Myc) were expressed along with mysterin-3FLAG in HEK293T cells. Cells were immunostained using anti-Myc (red) and anti-FLAG antibodies (green). The nucleus was stained using Hoechst 33342 (blue). Both isoforms were predominantly localized to the cytosol and co-localized with mysterin in all cells examined.

**Figure 3:**
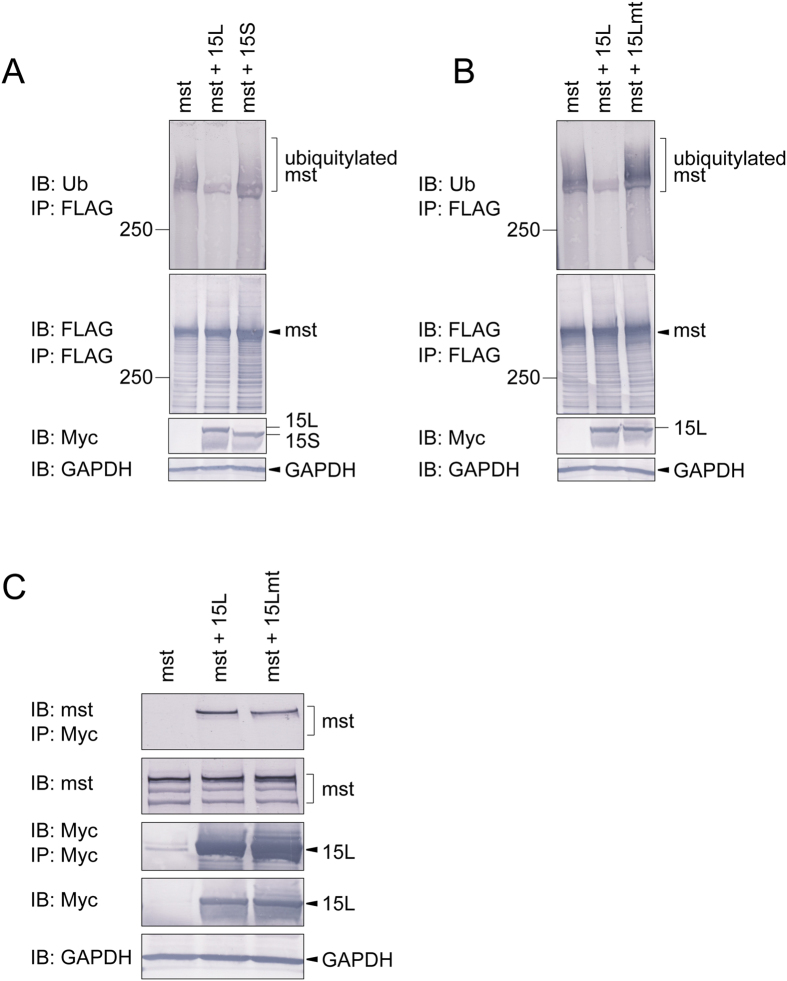
Mysterin is deubiquitylated predominantly by the long isoform of USP15. (**A**) Mysterin-3FLAG was expressed along with USP15L-Myc or USP15S-Myc in HEK293T cells, which were treated with proteasome inhibitor for 8 hours before harvest. The cell lysates were then subjected to immunoprecipitation using anti-FLAG affinity beads. The immunoprecipitate (i.e., crudely purified mysterin-3FLAG) was analyzed by immunoblotting of endogenous ubiquitin, revealing polyubiquitylation of mysterin (top panel), which was removed to a significant extent by USP15L. Lower panels show basal expression of the proteins and the internal marker GAPDH. Full-length blots are presented in Supplementary Fig. 4D. (**B**) Mysterin-3FLAG was expressed along with USP15L-Myc or enzymatic mutant USP15L-Myc (USP15Lmt-Myc) in HEK293T cells, which were treated with proteasome inhibitor for 8 hours before harvest, and then subjected to immunoprecipitation (anti-FLAG) and immunoblotting (anti-ubiquitin). USP15L deubiquitylated mysterin, whereas the enzymatic mutant (15 Lmt) increased polyubiquitylation to some extent. Full-length blots are presented in Supplementary Fig. 4E. (**C**) USP15L-Myc and USP15Lmt-Myc were transiently expressed and immunoprecipitated using anti-Myc antibody. Endogenous mysterin was co-immunoprecipitated to comparable extents with both isotypes (top panel), indicating that USP15Lmt retains mysterin-binding activity. Lower panels indicate basal expression levels of proteins and immunoprecipitation efficiency. Full-length blots are presented in Supplementary Fig. 4F.

**Figure 4:**
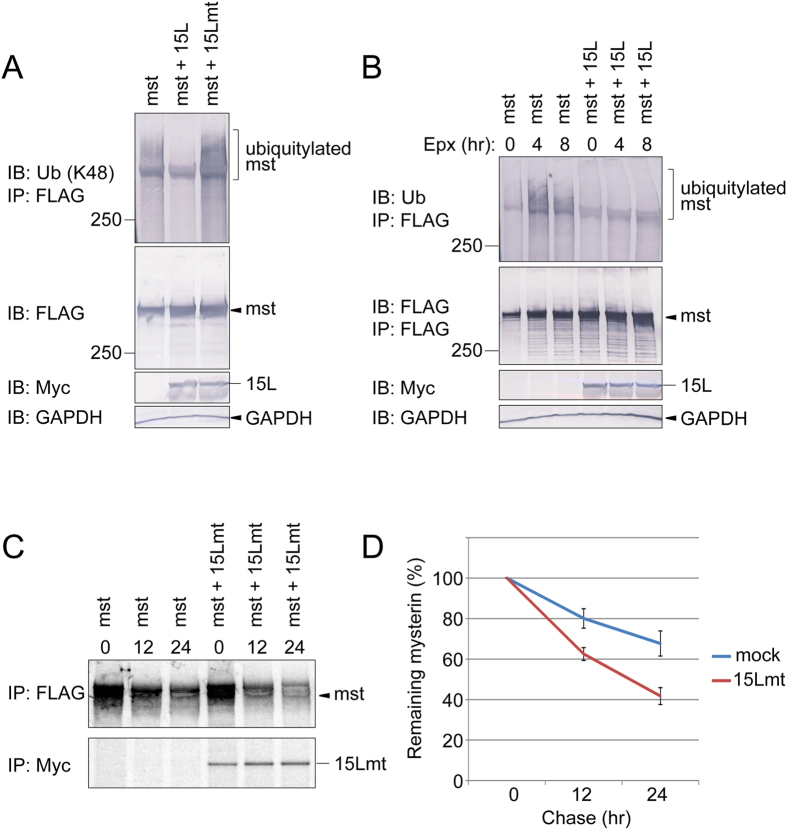
USP15L antagonizes proteasomal degradation of mysterin. (**A**) Mysterin-3FLAG was expressed along with USP15L-Myc or USP15Lmt-Myc in HEK293T cells, which were treated with proteasome inhibitor for 8 hours before harvest. The cell lysates were subjected to immunoprecipitation with anti-FLAG affinity beads and immunoblotting with anti-K48 linked polyubiquitin antibody to monitor the K48-linked polyubiquitylation status of mysterin. Lower panels show basal expression of the proteins and internal marker GAPDH. USP15L antagonized K48-linked ubiquitylation of mysterin, whereas the mutant increased polyubiquitylation. Full-length blots are presented in Supplementary Fig. 4G. (**B**) Mysterin-3FLAG was expressed with or without USP15L-Myc in HEK293T cells, which were treated with proteasome inhibitor for the indicated periods before harvest. Cell lysates were analyzed by immunoprecipitation using anti-FLAG affinity beads and immunoblotting using anti-K48 linked polyubiquitin antibody. Lower panels show basal expression of the proteins and the internal marker GAPDH. Proteasome inhibitor treatment resulted in accumulation of mysterin polyubiquitylation, which was antagonized by USL15L. Full-length blots are presented in Supplementary Fig. 4H. (**C**) HEK293T cells expressing mysterin-3FLAG with or without USP15Lmt-Myc were pulse-labeled with radioactive sulfur-containing cysteine and methionine for 1 hour, and then chased with non-radioactive medium for indicated periods. The radiolabeled mysterin-3FLAG was isolated by immunoprecipitation (anti-FLAG), separated by SDS-PAGE, and analyzed by autoradiography. Full-length autoradiographs are presented in Supplementary Fig. 4I. (**D**) Quantification of (**C**) USL15Lmt destabilized mysterin, presumably through a dominant-negative effect.

**Figure 5:**
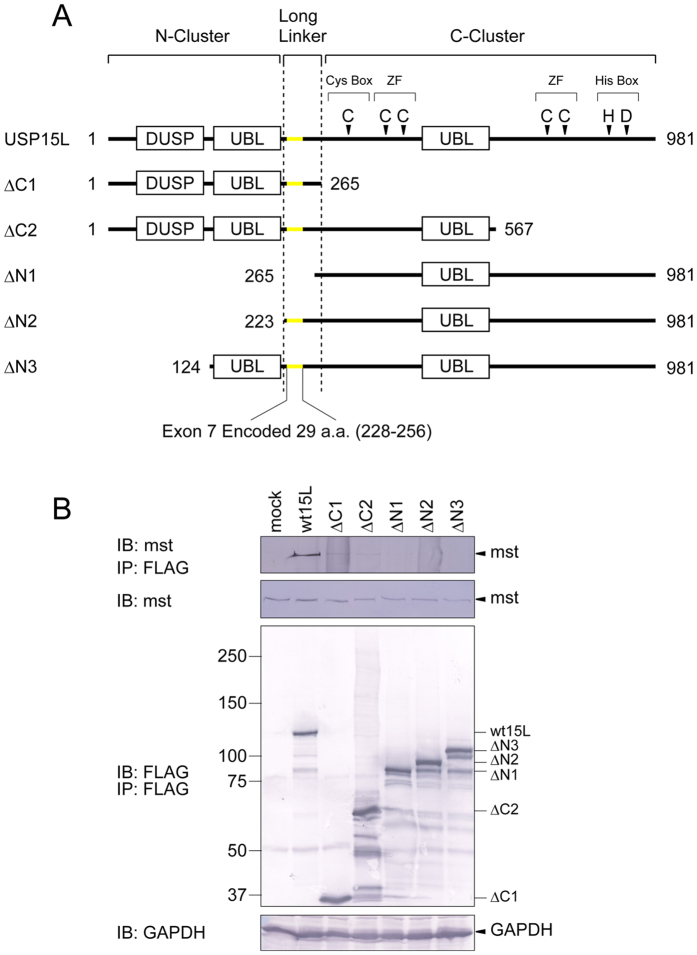
Domain(s) of USP15 responsible for mysterin recognition. (**A**) Architectures of deletion mutants of USP15L lacking N- or C-terminal regions as indicated. Full-length USP15L has a DUSP (domain present in USP), two UBLs (ubiquitin-like domains), a set of Cys and His boxes that constitutes the enzymatic core common throughout the USPs, and a zinc finger domain containing four ligand cysteines (ZF). (**B**) Wild-type and deletion mutants of USP15L-3FLAG (USP15L harboring three tandem FLAG tags at its C-terminus) were transiently expressed in HEK293T cells and isolated by precipitation using FLAG affinity beads. Endogenous co-precipitated mysterin was analyzed by immunoblotting using anti-mysterin antibody. None of the mutants exhibited significant affinity to endogenous mysterin. Full-length blots are presented in Supplementary Fig. 4J.

**Figure 6:**
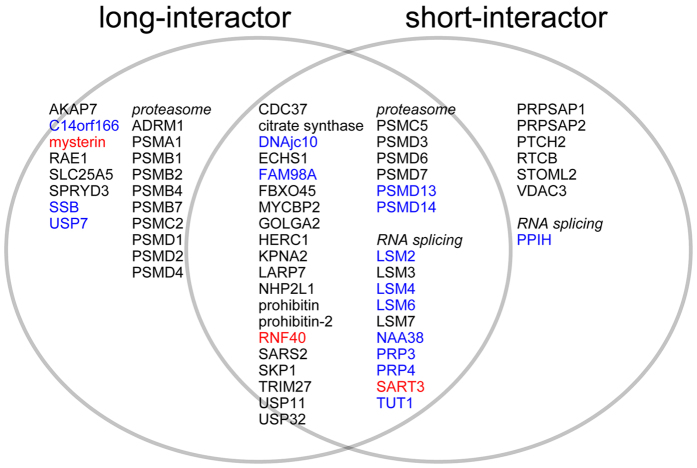
Interactome analysis of the long and short isoforms of USP15. Venn diagram depicting the interactomes of USP15L (left) and S (right), which partly overlap. All interactants were reproducibly identified in four MS analyses. Interactants previously identified by MS analysis are shown in blue, whereas those studied more deeply in current and previous work are shown in red. Mysterin was involved only in long-interactome. Some interactants are categorized as members of the same protein complex or functional network (e.g., proteasome subunits and RNA-splicing factors), and such proteins are grouped together here.

The exon 7-encoded short element in the linker region of USP15 was necessary but not sufficient for the physical association with mysterin. Likewise, the DUSP domain and the C-terminal region were also essential but not sufficient. The C-region harbors the enzymatic core responsible for deubiquitylation activity^12^,^13^, so the C-cluster presumably recognizes the ubiquitin moiety of the ubiquitylated mysterin. Previous structural studies demonstrated that the DUSP domain of USP15 exposes multiple hydrophobic patches on its molecular surface, which are predicted to mediate protein–protein interactions via hydrophobic interactions^29^,^30^. Indeed, USP15 specifically binds to SART3 (also known as Tip110) via DUSP domain and removes ubiquitin from both SART3 and its binding partner, histone H2B^9^,^27^,^28^. The N-terminal DUSP domain of USP15 may recognize the backbone polypeptide of mysterin in a similar manner. Mysterin and SART3, however, may be recognized by USP15 in different manners. Our interactome analysis demonstrated that SART3 is recognized by both isoforms, whereas only the long isoform exhibited significant binding to mysterin (Figs 2 and 6). Furthermore, the DUSP domain of USP15 is sufficient for binding to SART3^9^,^28^, although it is necessary but not sufficient for mysterin. The DUSP domain of USP15 may bind less strongly to mysterin than to SART3, creating a requirement for supportive binding by the C-terminal enzymatic region to the ubiquitin moiety. In this case, the inter-region linker could be involved in regulating the cooperative recognition by the N- and C-regions. To further explore this idea, determination of the overall structure of USP15 and further biochemical assessment are warranted.

Sixma and colleagues recently reported that the DUSP domain of USP4 promotes the deubiquitylation cycle by discharging hydrolyzed free ubiquitin from the enzymatic core of USP4 through inter-domain interactions^14^. However, the requirement for this activity was less important for USP15, and, as those authors discussed, it is possible that a single DUSP domain consecutively handles substrate recognition and ubiquitin release during the deubiquitylation reaction cycle.

Variation in the linker region is likely to have been maintained under natural selection. Phylogenic study suggested that USP4, 11, and 15 arose from a single ancestral USP, conserved between fungi and metazoans, following the emergence of vertebrates^4^. Although the ancestral USP, USP4, and USP15 retain exon 7, USP11 lacks it and constitutively generates only the short isoform. Experimental assessment identified a conserved nucleotide element in the 5′-terminal region of exon 7 of USP4 that specifically leads to insufficient splicing, resulting in the generation of the two alternative isoforms^17^. This mechanism is conserved at least among mammalians. The proactively generated short and long isoforms of USP4 exhibit clearly distinct subcellular distributions, supporting the hypothesis that this region is not a mere inter-region connection site. The functional relevance of the linker regions of USP4 and 15 could be related to their serine-rich compositions, which include multiple potential phosphorylation sites^4^,^17^. Specific regulation by serine kinases may occur throughout this region, although no evidence supporting this idea is currently available. Recent studies, including ours, suggest that variation in the inter-region linker is a key functional determinant for this class of USPs, although the molecular mechanism remains to be elucidated.

We initiated this study based on our interest in the function and/or regulation of the moyamoya disease susceptibility factor mysterin. The resultant data suggest that USP15L stabilizes mysterin via removal of K48-linked polyubiquitin chains. These observations were obtained under conventional cell culture conditions, suggesting that USP15L maintains the basal expression level of this ubiquitin ligase, which was previously shown to have autoubiquitylation (possibly, self-destabilizing) activity^19^, although it remains unclear whether the effect of USP15L on mysterin is also influenced by specific regulation or stimulation. Reduced mysterin expression causes abnormal organogenesis, including embryonic angiogenesis and myogenesis, in zebrafish^19^,^31^. Therefore, it is important to maintain the physiological expression level of mysterin, and the USP15 long isoform may contribute to this process *in vivo*. Despite the obvious phenotypes in mysterin knockdown/knockout zebrafish, the cellular and molecular functions of mysterin remain controversial. Recent studies proposed roles in insulin production in pancreatic β cells^32^, WNT signaling in endothelial cells^33^, and non-mitochondrial oxygen consumption in breast cancer cells^34^, although the underlying mechanism remains to be determined. How the suppression of mysterin expression causes abnormal organogenesis in zebrafish also remains completely unknown. Further investigation of these issues, as well as the potential involvement of USP15 in moyamoya disease, is warranted.

## Methods

### Plasmids

Full-length cDNAs for USP15L (NM_001252078.1) and USP15S (NM_006313.2) were obtained simultaneously by RT–PCR using the OneStep RT-PCR kit (QIAGEN, North Rhine-Westphalia, Germany) with template RNA extracted from HEK293 cells using the RNeasy Mini Kit (QIAGEN). USP15L or S with Myc or three tandem FLAG (3FLAG) epitope tag at the C-terminus was generated by PCR using forward primer USP15FNheI (ATGCGCTAGCACCATGGCGGAAGGCGGAGCGGC) and reverse primers USP15RMycNotI (ATGCGCGGCCGCCTACAGATCCTCTTCTGAGATGAGTTTTTGTTCGTTAGTGTGCATACAG) or USP15R3FLAGHindIII (ATGCAAGCTTTTACTTATCGTCGTCATCCTTGTAGTCGATGTCATGGTCTTTGTAGTCACCGTCATGATCCTTGTAATCGTTAGTGTGCATACAGTTTTC). These PCR products, harboring exogenous restriction sites and epitope tag at the 3′ terminus, were subcloned using *Nhe*I/*Not*I sites (Myc) or *Nhe*I/*Hin*dIII sites (3FLAG) into the mammalian expression vector pcDNA3.1+ (Invitrogen, CA, USA). Restriction enzymes were purchased from New England Biolab (MA, USA) or Fermentas (MA, USA). DNA ligase was purchased from Takara (Shiga, Japan). The enzymatic mutant of USP15L (15Lmt), which harbors alanines instead of the cysteines at positions 269 and 812, and that of USP15S (15Smt), which harbors alanine instead of the cysteine at positions 269, were generated by mutagenesis PCR using the QuikChange Site-Directed Mutagenesis kit (Agilent Technologies, CA, USA) using primers USP15C298AF (GCCTCTGTGGCCTAAGTAACTTGGGAAATACGGCTTTCATGAACTCAGCTATTCAGTGTTTGAGCAACACACC), USP15C298AR (GGTGTGTTGCTCAAACACTGAATAGCTGAGTTCATGAAAGCCGTATTTCCCAAGTTACTTAGGCCACAGAGGC), USP15C812AF (GCTGAAGATCCCTGGTATTGTCCGAATGCTAAAGAACATCAGCAAGCCACAAAGAAATTGG), and USP15C812AR (CCAATTTCTTTGTGGCTTGCTGATGTTCTTTAGCATTCGGACAATACCAGGGATCTTCAGC). Deletion mutants were generated using seven primers USP15FNheI (described above), USP15F2NheI (ATGCGCTAGCATGCCATCATATACCGCTTATAAGAAC), USP15FdN2NheI (ATGCGCTAGCCGCCATGGGTCCTTCTACTCCTAAGTCC), USP15FdN3NheI (ATGCGCTAGCCGCCATGGTAAAGCACTGCAAAGTAGAA), USP15R13FLAGHindIII (ATGCAAGCTTCTACTTATCGTCGTCATCCTTGTAGTCGATGTCATGGTCTTTGTAGTCACCGTCATGATCCTTGTAATCAAGACAGTAATTTGAGTTTTTCACATT), USP15R23FLAGHindIII (ATGCAAGCTTCTACTTATCGTCGTCATCCTTGTAGTCGATGTCATGGTCTTTGTAGTCACCGTCATGATCCTTGTAATCAATAATCACGTGCTCTGTATCTTCTGTCC), and USP15R33FLAGHindIII (ATGCAAGCTTTTACTTATCGTCGTCATCCTTGTAGTCGATGTCATGGTCTTTGTAGTCACCGTCATGATCCTTGTAATCGTTAGTGTGCATACAGTTTTC). ΔC1, ΔC2, ΔN1, ΔN2, and ΔN3 deletion mutants were generated by PCR using USP15FNheI and USP15R13FLAGHindIII, USP15FNheI and USP15R23FLAGHindIII, USP15F2NheI and USP15R33FLAGHindIII, USP15FdN2NheI and USP15R33FLAGHindIII, and USP15FdN3NheI and USP15R33FLAGHindIII, respectively.

Mysterin-FLAG and -3FLAG were generated in our previous studies^19^,^22^. EGFP-hnRNPK was a kind gift from Dr. Kenji Irie (University of Tsukuba, Ibaragi, Japan)^35^. HA-ubiquitin was described previously^36^. Full-length cDNAs of IP3R3 and LYAR were cloned using methods and materials similar to those used for the cloning of USP15L/S. IP3R3 was initially amplified as five fragments, which were ligated using intrinsic restriction sites *Kpn*I, *Not*I, *Sal*I, and *Bam*HI. Exogenous *Nhe*I and *Xba*I sites and the Myc epitope sequence were introduced into the 5′ and 3′ termini by PCR using forward primer CloNheIIP3R3F1 (ATGCGCTAGCCGCCATGAGTGAAATGTCCAGCTTTCTTCACATCGG) and reverse primer CloXbaIIP3R3R53FLAG (TACGTCTAGATCAGATGTCATGATCTTTATAATCACCGTCATGGTCTTTGTAGTCCTTATCGTCGTCATCCTTGTAATCGCGGCTAATGCAGTTCTGG). The PCR product was subcloned into pcDNA3.1+ using the *Nhe*I/*Xba*I sites. Full-length cDNA of LYAR was obtained by RT-PCR using forward primer KpnILYARCloF (ATGCGGTACCGCCACCATGGTATTTTTTACATGC) and reverse primer NotI3FLAGLYARCloR (TACGGCGGCCGCTTAGATGTCATGATCTTTATAATCACCGTCATGGTCTTTGTAGTCCTTATCGTCGTCATCCTTGTAATCTTTCACAAGCTTGACTTTG), and then subcloned into pcDNA3.1+ using the *Kpn*I/*Not*I sites.

### Cell culture and transfection

HEK293 (used only for cloning of USP15) and HEK293T cells were maintained in DMEM (Life Technologies, California, USA) containing 10% fetal bovine serum, 100 U/mL penicillin, and 100 μg/mL streptomycin. Plasmids were transfected into HEK293T cells using Lipofectamine LTX (Life Technologies) or PEI MAX (Polysciences, Pennsylvania, USA). The amount of DNA was adjusted to obtain equivalent expression levels of the introduced proteins in each experiment. For detection of ubiquitylation, cells were treated with 1 μM epoxomicin (Peptide Institute, Osaka, Japan) for 8 h (or other indicated period) before harvest.

### Antibodies

Antibodies used for immunoprecipitation (IP), immunoblotting (IB), or immunostaining (IS) in this study include anti-FLAG antibody (M2 used for IP and IB, Sigma Aldrich, St. Louis, MO, USA), anti-Myc antibody (rabbit polyclonal for IS, MBL, Aichi, Japan; 9E10 for IP and IB, Santa Cruz Biotechnology, Dallas, TX, USA), andi-HA antibody (6E2 for IB, Cell Signaling, MA, USA), anti-ubiquitin antibody (FK2 for IB, Enzo Life Sciences, NY, USA), anti-K48 linked ubiquitin antibody (Apu2 for IB, Enzo Life Sciences), and anti-GAPDH antibody (6C5 for IB, Hy Test Ltd, Turku, Finland). Anti-human mysterin mouse monoclonal antibody (1C9) was generated with the method previously reported^37^. Briefly, male BALB/C mice were immunized by inoculations of 200 μg recombinant partial human mysterin protein (amino acids 1–349), which was obtained by affinity purification using the C-terminal FLAG epitope tag. The mice were boosted with additional recombinant protein 4 days before fusion. Spleen cells were fused with a myeloma cell line. Following fusion, clones were evaluated by ELISA and immunoblotting using mysterin antigen.

### LC-MS/MS

Mysterin-FLAG or USP15L/S-3FLAG was expressed in HEK293T cells, and associated proteins were recovered from cell extracts by immunoprecipitation with anti-FLAG antibody. The associated protein complexes with mysterin-FLAG were immediately digested with lysyl endopeptidase (Lys-C, Wako, Osaka, Japan). Protein complexes associated with USP15L/S-FLAG were reduced with 5 mM TCEP, and then alkylated with 10 mM monoiodoacetoamide. The alkylated protein complexes were digested with Lys-C and trypsin (SCIEX, Tokyo, Japan). The resultant peptides were analyzed using a direct nano-flow LC-MS/MS system, as described previously^38^.

### Immunoprecipitation

Protein-encoding or empty vector was transiently transfected into HEK293T cells. The cells were harvested 24 or 48 hours after the transfection and lysed with HEPES buffer (150 mM NaCl, 20 mM HEPES, NaOH, pH 7.4) containing 1% NP40 (for ubiquitylation assay) or digitonin (for all other experiments). The lysate was centrifuged at 13,000 *g*, and the supernatant was incubated for 1 hour with anti-FLAG M2 agarose beads (Sigma Aldrich) or anti-Myc antibody and protein G beads (GE Healthcare, Buckinghamshire, UK). At this point, aliquots of supernatant were resolved with Laemmli’s sample buffer containing 1 mM DTT as a control (i.e., representing sample not subjected to immunoprecipitation). The beads were washed three times with digitonin-containing HEPES buffer and resolved with Laemmli’s sample buffer containing 1 mM DTT.

### Immunoblotting

Samples were separated with SDS-PAGE using 7.5% or 3–10% acrylamide gel (ATTO, Tokyo, Japan) and transferred to nitrocellulose (GE Healthcare) or PVDF (Merck Millipore, Hessen, Germany) membrane for 1.5 hours at 75 V at 4 °C. Membrane was blocked using Blocking One (Nacalai Tesque, Kyoto, Japan) for 1 hour at room temperature, and then incubated sequentially with primary and secondary antibodies diluted in Can Get Signal (TOYOBO, Tokyo, Japan). Detection was performed via the chemiluminescence or the alkaline phosphatase method.

### Immunostaining

HEK293T cells expressing USP15L-Myc or S-Myc together with mysterin-3FLAG were immobilized with 4% paraformaldehyde (Nacalai Tesque) for 10 min at room temperature 24 hours after transfection. The cells were permeabilized with 100 ng/mL digitonin for 5 minutes, and then washed with phosphate-buffered saline (PBS: 137 mM NaCl, 2.68 mM KCl, 8.1 mM Na_2_HPO_4_ and 1.47 mM KH_2_PO_4_, pH 7.4). After incubation with Blocking One (Nacalai Tesque) for 4 hours at room temperature, the cells were incubated sequentially with primary and fluorescence-conjugated second antibodies; each of the antibody incubations were followed by PBS washes. The nucleus was stained using Hoechst 33342. Fluorescence images were obtained by confocal microscopy (LSM 700, Zeiss, Oberkochen, Germany).

### Pulse chase analysis

Pulse chase experiment was performed with the method previously described^36^. HEK293T cells expressing mysterin-3FLAG with or without USP15Lmt-Myc or USP15Smt-Myc) were pulse-labeled with 4.1 MBq/3.5 cm dish of EXPRESS labeling mixture (PerkinElmer Life and Analytical Sciences, Massachusetts, USA) in methionine- and cysteine-free DMEM supplemented with 2 mM glutamine and 10% dialyzed fetal bovine serum, and then chased with fresh complete medium for indicated time periods. Cells were lysed in HEPES buffer containing 1% NP-40, and supernatants were incubated with M2 affinity beads (Sigma Aldrich) or anti-Myc antibody and protein G beads (GE Healthcare). The beads were washed twice in the buffer used to lyse the cells, and then incubated in Laemmli’s sample buffer. Immunoprecipitates were separated by SDS-PAGE, and radioactivity of specific bands was quantified using a Storm PhosphorImager (GE Healthcare). Quantification and data processing was performed using the ImageJ software (NIH, USA) and Microsoft Excel.

## Additional Information

**How to cite this article:** Kotani, Y. *et al*. Alternative exon skipping biases substrate preference of the deubiquitylase USP15 for mysterin/RNF213, the moyamoya disease susceptibility factor. *Sci. Rep.* **7**, 44293; doi: 10.1038/srep44293 (2017).

**Publisher's note:** Springer Nature remains neutral with regard to jurisdictional claims in published maps and institutional affiliations.

## Supplementary Material

Supplementary Figures
