# Supplementary material for: Alternative exon skipping biases substrate preference of the deubiquitylase USP15 for mysterin/RNF213, the moyamoya disease susceptibility factor
Source: Sci Rep. 2017 Mar 9;7:44293. doi: 10.1038/srep44293 (PMC5343593; doi:10.1038/srep44293)

Supplementary Information

Alternative exon skipping biases substrate preference of the deubiquitylase USP15 for mysterin/RNF213, the moyamoya disease susceptibility factor

Yuri Kotani[1], Daisuke Morito[2,3,*], Kenshiro Sakata[1], Shiori Ainuki[1], Munechika Sugihara[1], Tomohisa Hatta[4], Shun-ichiro Iemura[4], Seiji Takashima[5], Tohru Natsume[4], Kazuhiro Nagata[1,2,3]

[1] Faculty of Life Sciences, Kyoto Sangyo University, 603-8555, Japan
[2] Institute for Protein Dynamics, Kyoto Sangyo University, 603-8555, Japan
[3] CREST, Japan Science and Technology Agency, 332-0012, Japan
[4] Biomedicinal Information Research Center, National Institute of Advanced Industrial Science and Technology, 135-0064, Japan
[5] Graduate School of Medicine, Osaka University, 565-0871, Japan

*Corresponding author: Daisuke Morito (morito@cc.kyoto-su.ac.jp)

Supplementary Figure Legends

Supplementary SFig. 1. Potential nuclear localization of USP15S

The short isoform of USP15 harboring Myc epitope tag at their C-terminus (USP15S-Myc) was expressed along with mysterin-3FLAG in HEK293T cells. Cells were immunostained using anti-Myc (red) and anti-FLAG antibodies (green). The nucleus was stained using Hoechst 33342 (blue). Although most cells showed exclusive cytosolic distribution of USP15S (See Fig. 2E), a few cells showed additional faint distribution of USP15S in the nucleus.

Supplementary SFig. 2. Polyubiquitylation assay after denaturation treatment

HEK293T cells expressing ubiquitin harboring HA epitope tag at its N-terminus (HA-ubiquitin) with or without mysterin-3FLAG and USP15L-Myc were lysed 8 hours after the treatment with proteasome inhibitor and incubated for 20 minutes at 37 degree C in the presence of 0.5% SDS to dissociate potential non-covalent binding between mysterin and some proteins. The lysate was diluted 10-fold and subjected to immunoprecipitation with anti-FLAG affinity agarose and immunoblot with anti-HA antibody. Polyubiquitin was still associated with mysterin, indicating that the polyubiquitin signal is not due to potential mysterin binding protein which should be dissociated by incubation with 0.5% SDS before the immunoprecipitation but is due to mysterin *per se*.

Supplementary SFig. 3. USP15S does not affect the stability of mysterin

(**A**) HEK293T cells expressing mysterin-3FLAG with or without USP15Smt-Myc were pulse-labeled with radioactive sulfur-containing cysteine and methionine for 1 hour, and then chased with non-radioactive medium for indicated periods. The radiolabeled mysterin-3FLAG and USP15Smt-Myc were isolated by immunoprecipitation (anti-FLAG or anti-Myc), separated by SDS-PAGE, and analyzed by autoradiography. (**B**) Quantification of (A). USL15Smt did not affect mysterin in contrast to the obvious destabilization effect of USP15Lmt (See Fig. 4C and D).

Supplementary SFig. 4. Full-length blots and autoradiographs before cropping

(A) Original blots for Fig. 2A. (B) Original blots for Fig. 2B. (C) Original blots for Fig. 2C. (D) Original blots for Fig. 3A. (E) Original blots for Fig. 3B. (F) Original blots for Fig. 2C. (G) Original blots for Fig. 4A. (H) Original blots for Fig. 4B. (I) Original autoradiographs for Fig. 4C. (J) Original blots for Fig. 5B.

Kotani et al. Supplementary SFig. 1

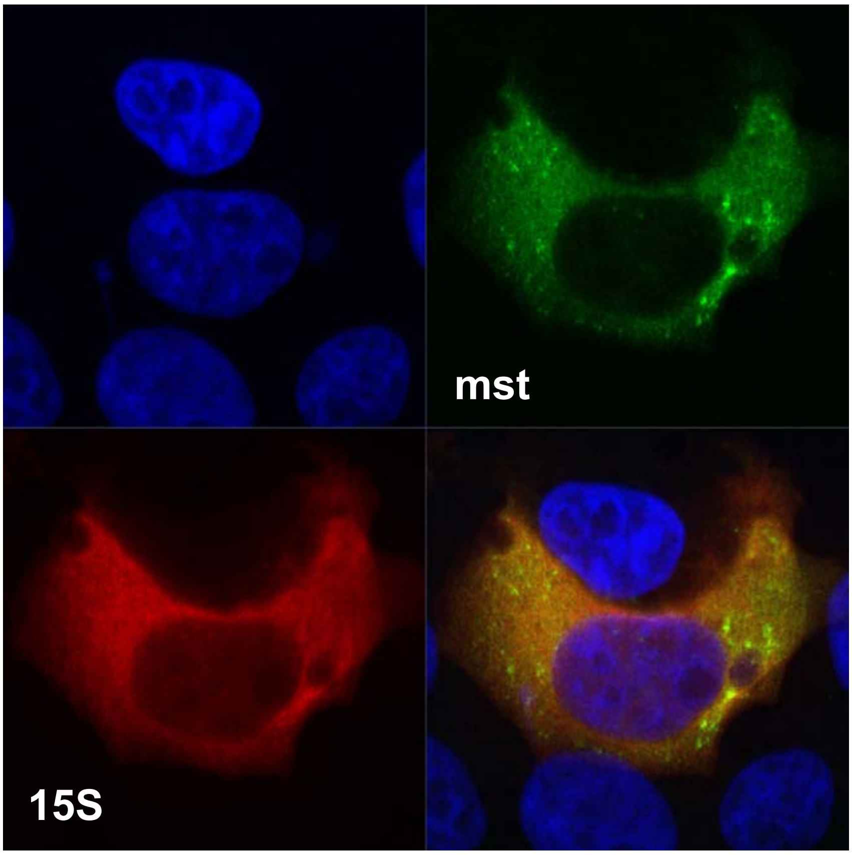

Kotani et al. Supplementary SFig. 2

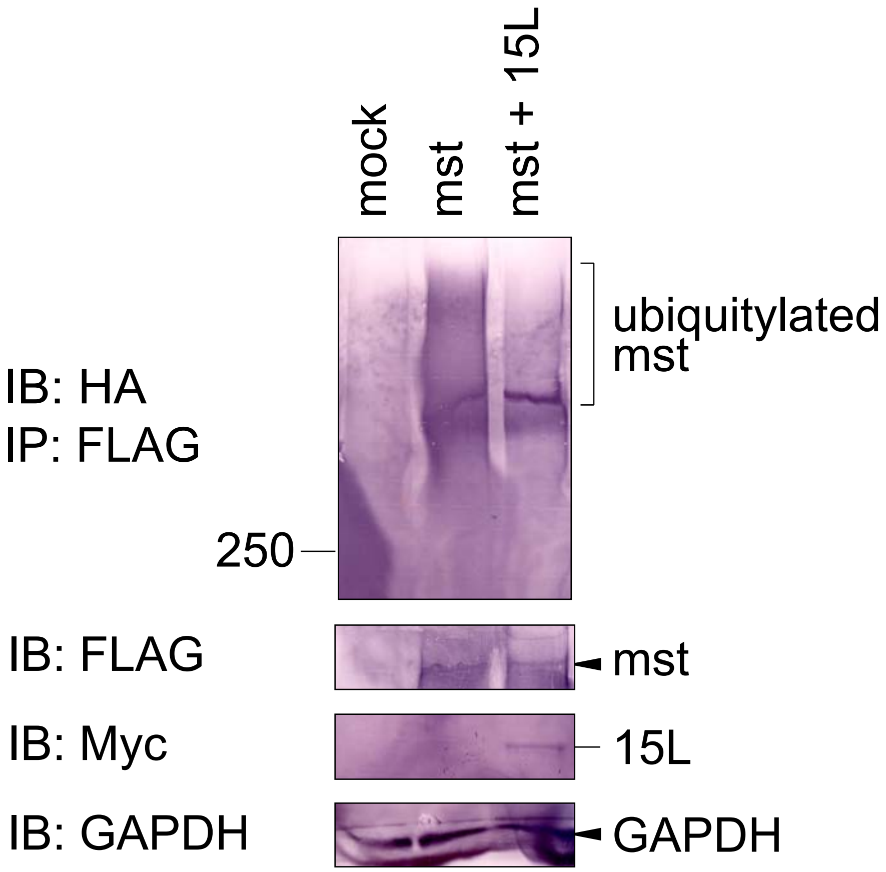

Kotani et al. Supplementary SFig. 3

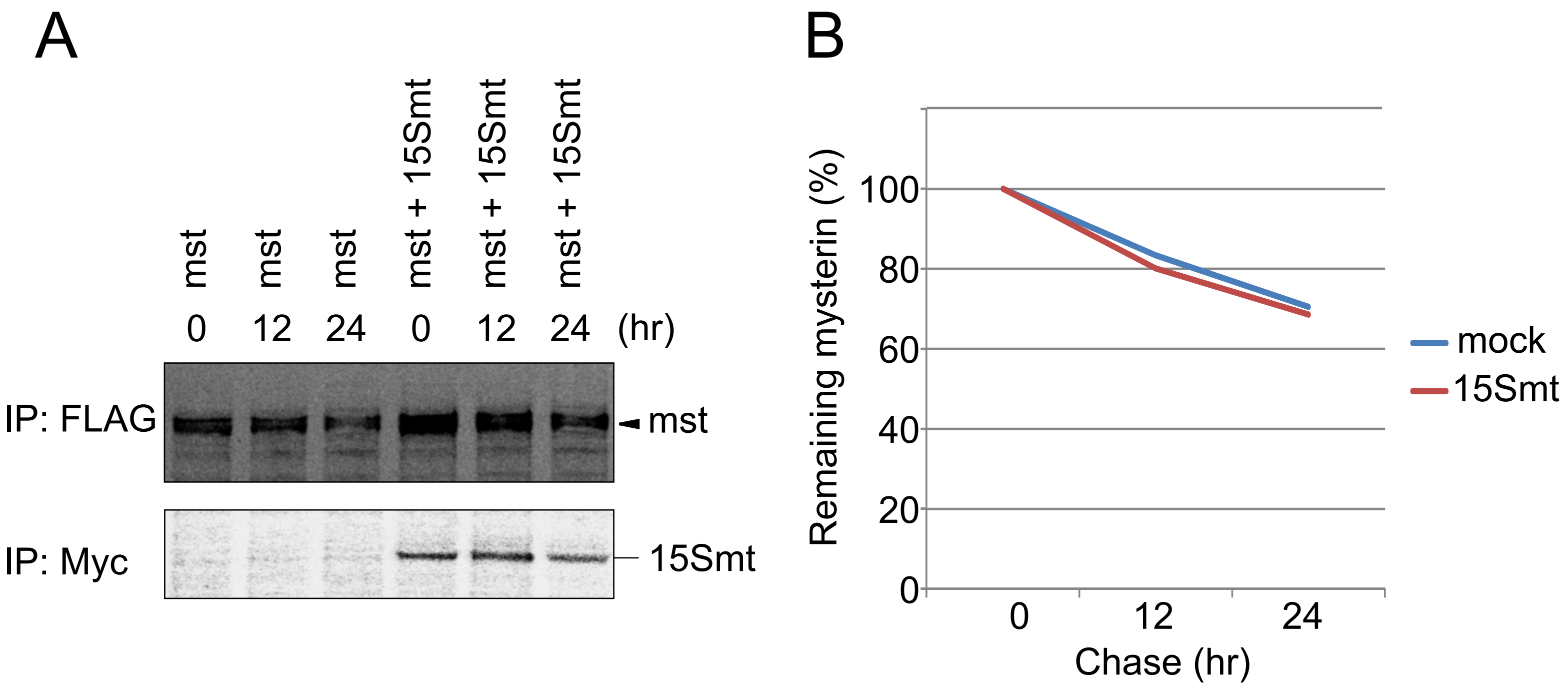

Kotani et al. Supplementary SFig. 4

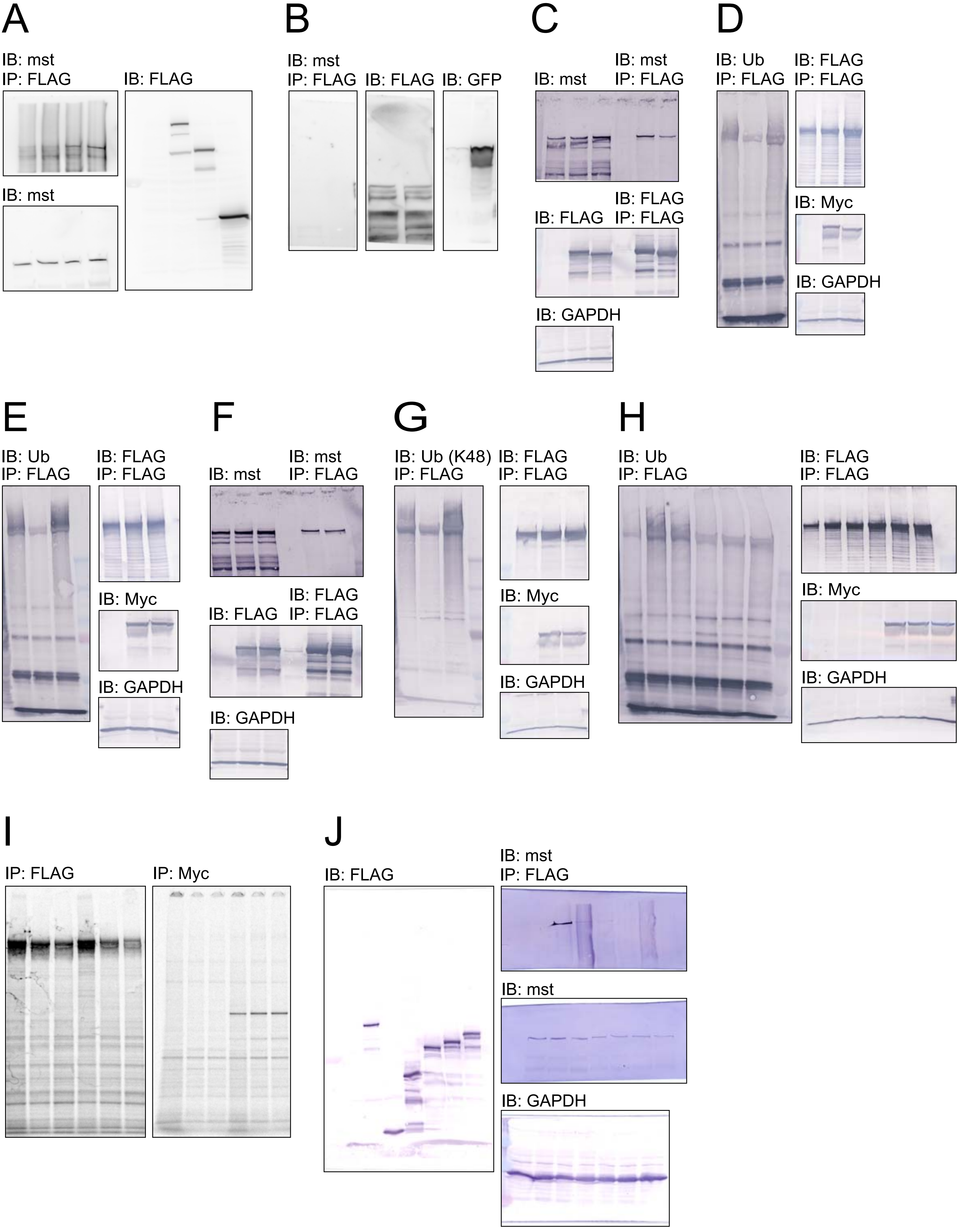

Supplement: Supplementary Figures [file srep44293-s1.pdf]
